# Supplementary material for: Mesenchymal-epithelial crosstalk shapes intestinal regionalisation via Wnt and Shh signalling
Source: Nat Commun. 2022 Feb 7;13:715. doi: 10.1038/s41467-022-28369-7 (PMC8821716; doi:10.1038/s41467-022-28369-7)
Supplement: Supplementary file 1 — Supplementary Information [file 41467_2022_28369_MOESM1_ESM.docx]

Supplementary Information File

Mesenchymal-epithelial crosstalk shapes intestinal regionalisation via Wnt and Shh signalling

Martti Maimets^1,2,3 #^, Marianne Terndrup Pedersen^1,2, #^, Jordi Guiu^1,2^, Jes Dreier^2,4^, Malte Thodberg^1,2,5^, Yasuko Antoku^1^, Pawel J. Schweiger^1,2^, Leonor Rib^1^, Raul Bardini Bressan^1,2,3^, Yi Miao^6^, K. Christopher Garcia^6,7^, Albin Sandelin^1,5^, Palle Serup^2,3^ and Kim B. Jensen^1,2,3*^

1- Biotech Research and Innovation Centre (BRIC), University of Copenhagen, Denmark

2- The Novo Nordisk Foundation Center for Stem Cell Biology (DanStem), University of Copenhagen, Denmark

3- The Novo Nordisk Foundation Center for Stem Cell Medicine (reNEW), University of Copenhagen, Denmark

4- The Novo Nordisk Foundation Center for Protein Research, CPR, University of Copenhagen, Denmark

5- The Bioinformatics Centre, Department of Biology, University of Copenhagen, Denmark

6- Department of Molecular and Cellular Physiology, Howard Hughes Medical Institute, Stanford University School of Medicine, Stanford, CA 94305, USA

7- Department of Structural Biology, Howard Hughes Medical Institute, Stanford University School of Medicine, Stanford, CA 94305, USA

#- These authors contributed equally

*- email: kim.jensen@sund.ku.dk

**This file includes:**

**Supplementary Figure 1.** Characterization of mesenchymal subpopulations in fetal intestine

**Supplementary Figure 2.** Regionalisation patterns in fetal intestinal cells

**Supplementary Figure 3.** Expression patterns of Wnt pathway members in fetal intestine

**Supplementary Figure 4.** Strategy and validation of TCF/LEF reporter model

**Supplementary Figure 5.** Characterization of the effects of PORCNi during fetal development

**Supplementary Figure 6.** Strategy for quantification of morphological features in the intestine

**Supplementary Figure 7.** Shh is a direct target for Wnt signalling

**Supplementary table 1:** Distances measured between epithelium and muscle

**Supplementary table 2:** Overlap of Wnt-dependent distal genes

**Supplementary table 3:** List of antibodies used

**
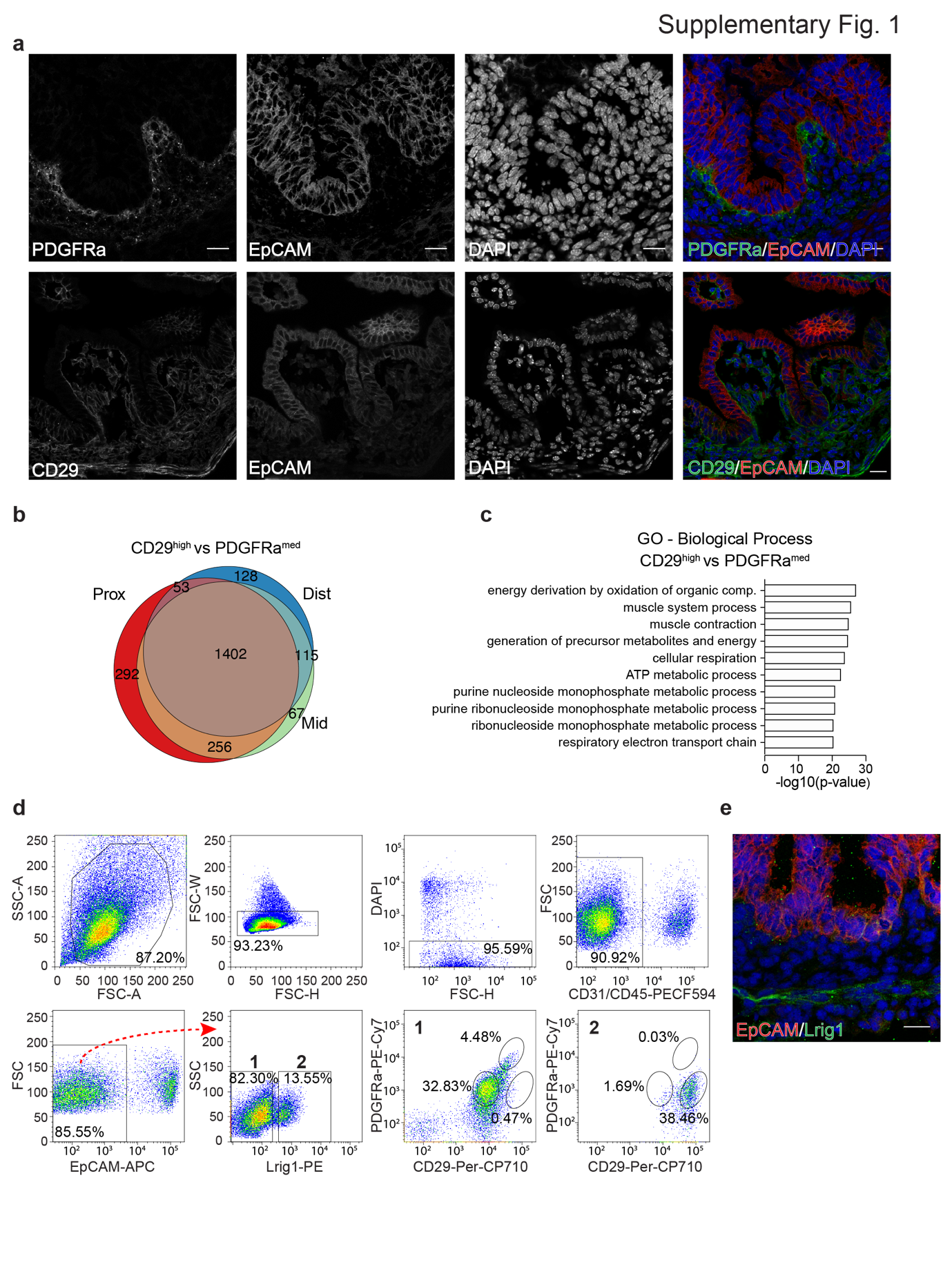
**

**Supplementary Fig. 1: Characterization of mesenchymal subpopulations in fetal intestine. a,** Detection of EpCAM, DAPI and PDGFRa (upper row) or CD29 (lower row) at E16.5. Scale bar, 20 μm. **b,** Euler diagram showing the overlap of up-regulated genes in CD29^high^ versus PDGFRa^med^ proximal, mid and distal regions. **c,** GO-term enrichment analysis showing top 10 terms in the Biological Process category for the gene set upregulated in CD29^high^ versus PDGFRa^med^ populations across all regions (overlap in panel b). **d**, Strategy for FACS analyses and the profile showing high expression of LRIG1 specifically in the CD29^high^/PDGFRa^med^ population. **e,** Detection of EpCAM (red), DAPI (blue) and LRIG1 (green) at E16.5. Scale bar, 20 μm. Data in a, d and e are representative of n = 3 independent experiments.

**
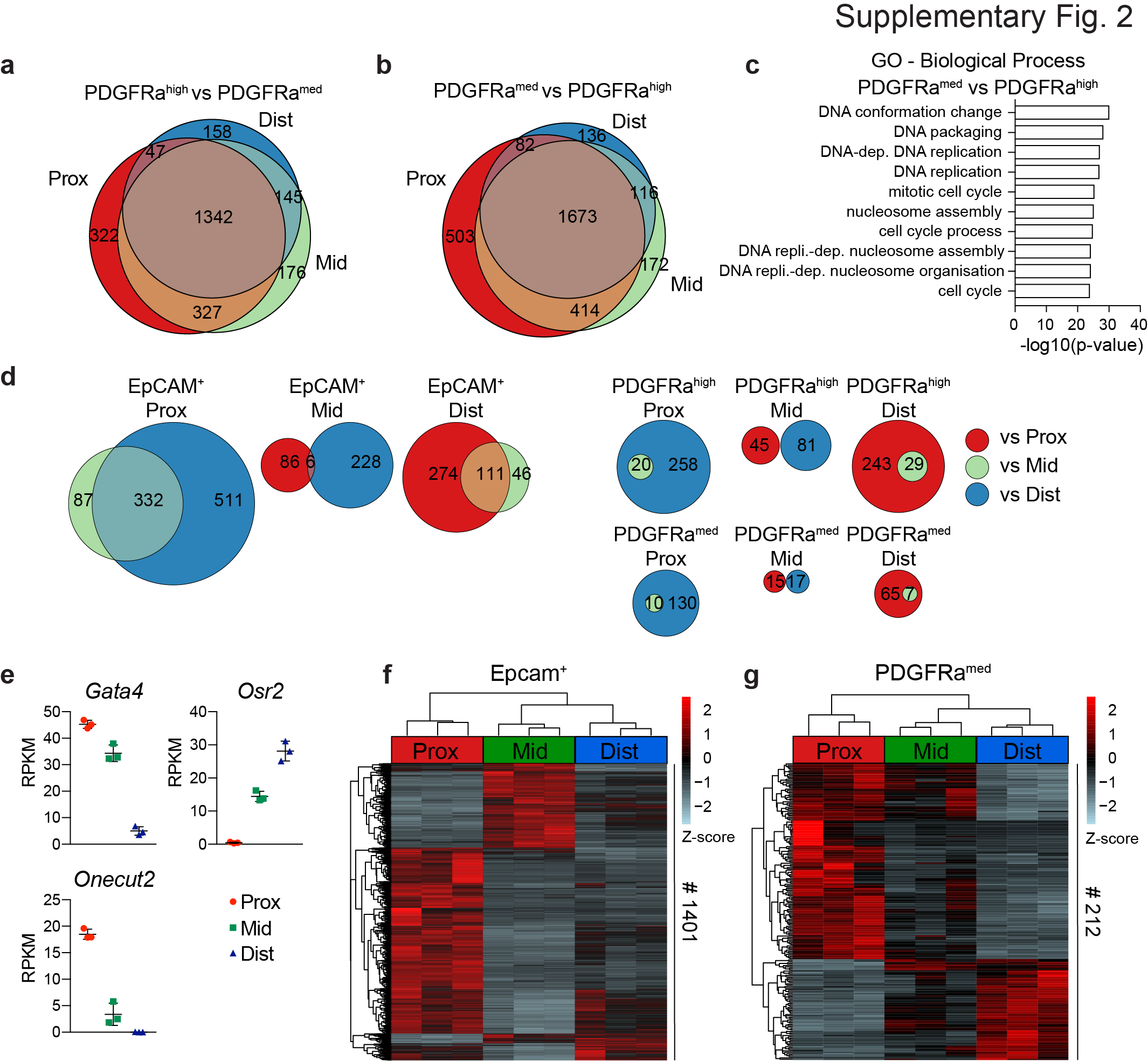
**

**Supplementary Fig. 2: Regionalisation patterns in fetal intestinal cells. a,** Euler diagram showing the overlap of up-regulated genes in PDGFRa^high^ versus PDGFRa^med^ proximal, mid and distal regions. **b,** Euler diagram showing the overlap of up-regulated genes in PDGFRa^med^ versus PDGFRa^high^ proximal, mid and distal regions. **c,** GO-term enrichment analysis showing top 10 terms in the Biological Process category for the gene set upregulated in PDGFRa^med^ versus PDGFRa^high^ populations across all regions (overlap in panel b). **d,** Euler diagrams showing the number of differentially expressed genes comparing proximal, mid and distal regions for EpCAM^+^ cells (left), PDGFRa^high^ (top row, right), and PDGFRa^med^ (bottom row). **e,** *Gata4, Onecut2,* and *Osr2* expression levels in RNA-seq analyses of EpCAM^+^ cells. The y-axis shows RPKM (reads per kilobase per million mapped reads). **f-g,** Heatmaps showing differentially expressed genes for **f)** EpCAM^+^ and **g)** PDGFRa^med^ cells comparing proximal, mid and distal regions. All panels present RNA-seq data from n = 3 biological replicates. Graphs in e) show means ± s.d.

**
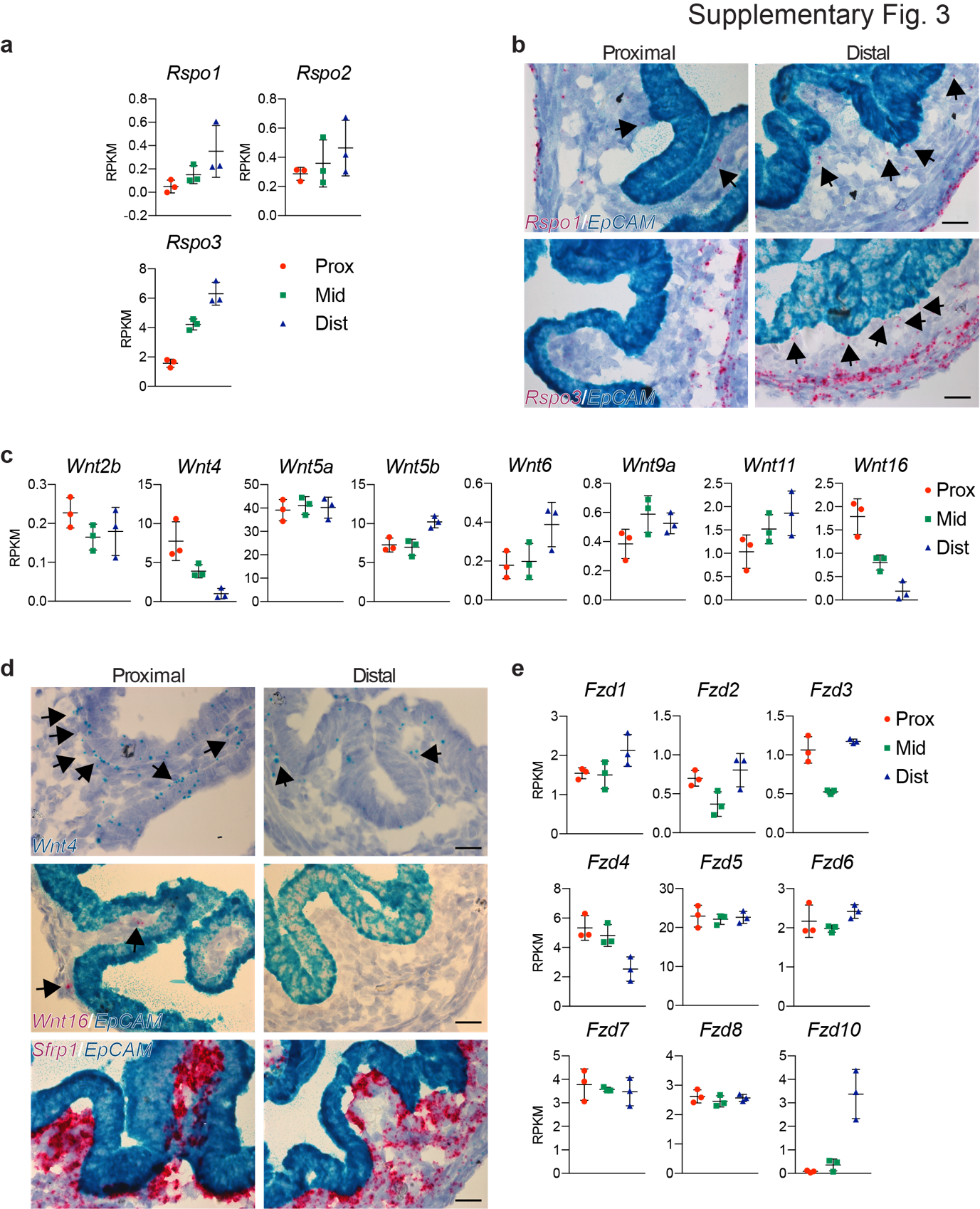
**

**Supplementary Fig. 3: Expression patterns of Wnt pathway members in fetal intestine.** **a,** RNA-seq analysis of *Rspo* family members detected in PDGFRa^high^ samples. **b,** In situ hybridisation for *EpCAM* (blue) and *Rspo1* (red, upper row) or *Rspo3* (red, lower row) on proximal and distal SI regions at E16.5. Scale bar, 20 μm. **c,** RNA-seq analysis of *Wnt* family members detected in PDGFRa^high^ samples across all replicates. **d,** In situ hybridisation for *Wnt4* (blue, upper row) or *EpCAM* (blue, middle and lower row) and *Wnt16* (red, middle row) or *Sfrp1* (red, lower row) on proximal and distal SI regions at E16.5. Scale bar, 20 μm. **e,** RNA-seq analysis of *Fzd* family members detected in EpCAM^+^ samples. Note that only *Rspo1, Rspo3, Wnt4, Wnt16, Fzd4,* and *Fzd10* are called as significantly differentially expressed comparing proximal and distal samples (log2(fold change) > 0.5 and FDR < 0.05). Data presented in a, c and e are means ± s.d. from n = 3 biological replicates. Results in b and d are representative of n = 2 biological replicates.


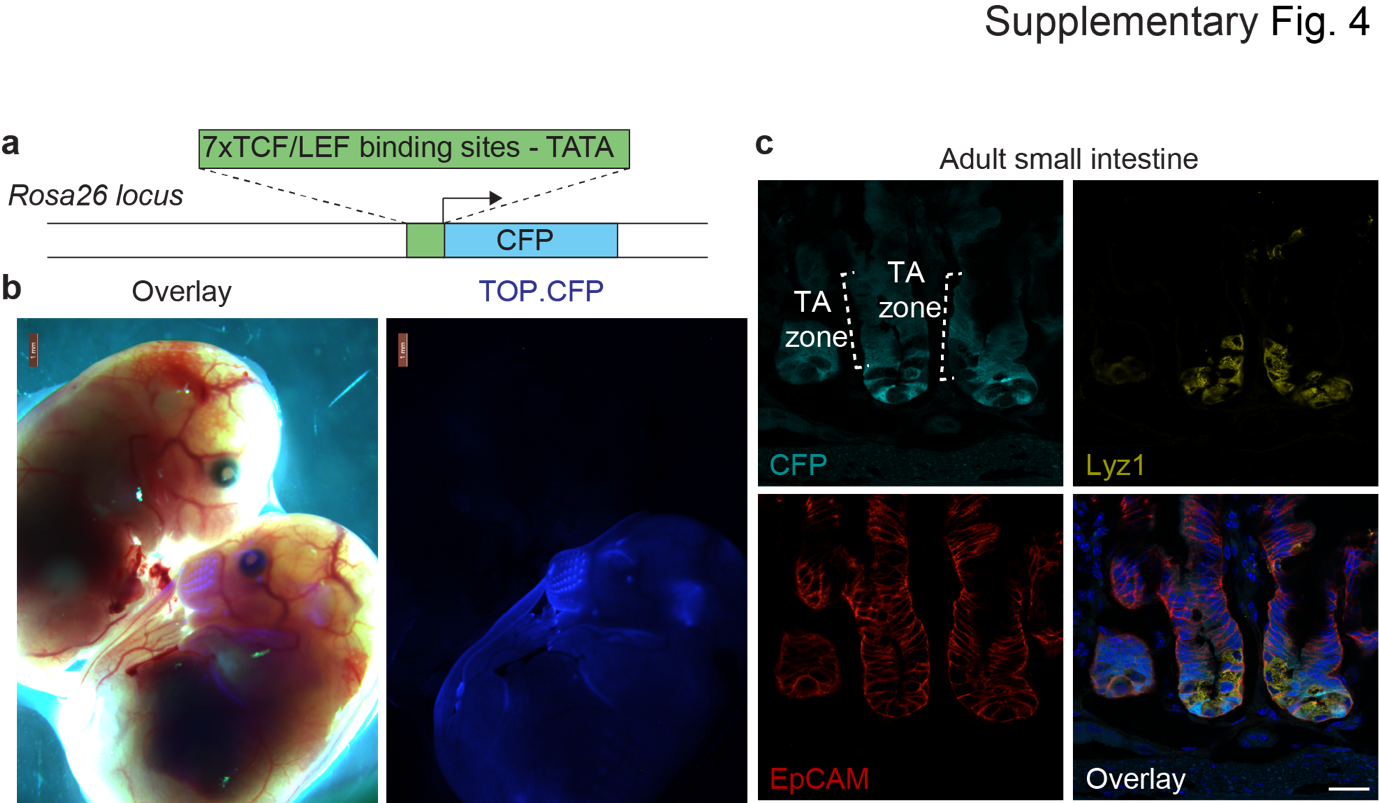


**Supplementary Fig. 4: Strategy and validation of TCF/LEF reporter model. a,** Graphical depiction of the TCF/LEF response reporter. **b,** E16.5 littermate embryos without (left) and with the Top.CFP transgene (right). Left panel, overlay of brightfield and CFP. Right panel, CFP. Scale bar, 1 mm. **c,** Detection of Top.CFP (cyan), LYZ1 (yellow) and EpCAM (red) in adult small intestine. Scale bar, 20 μm. TA: Transient amplifying. Data in b and c are representative of n = 3 independent experiments.


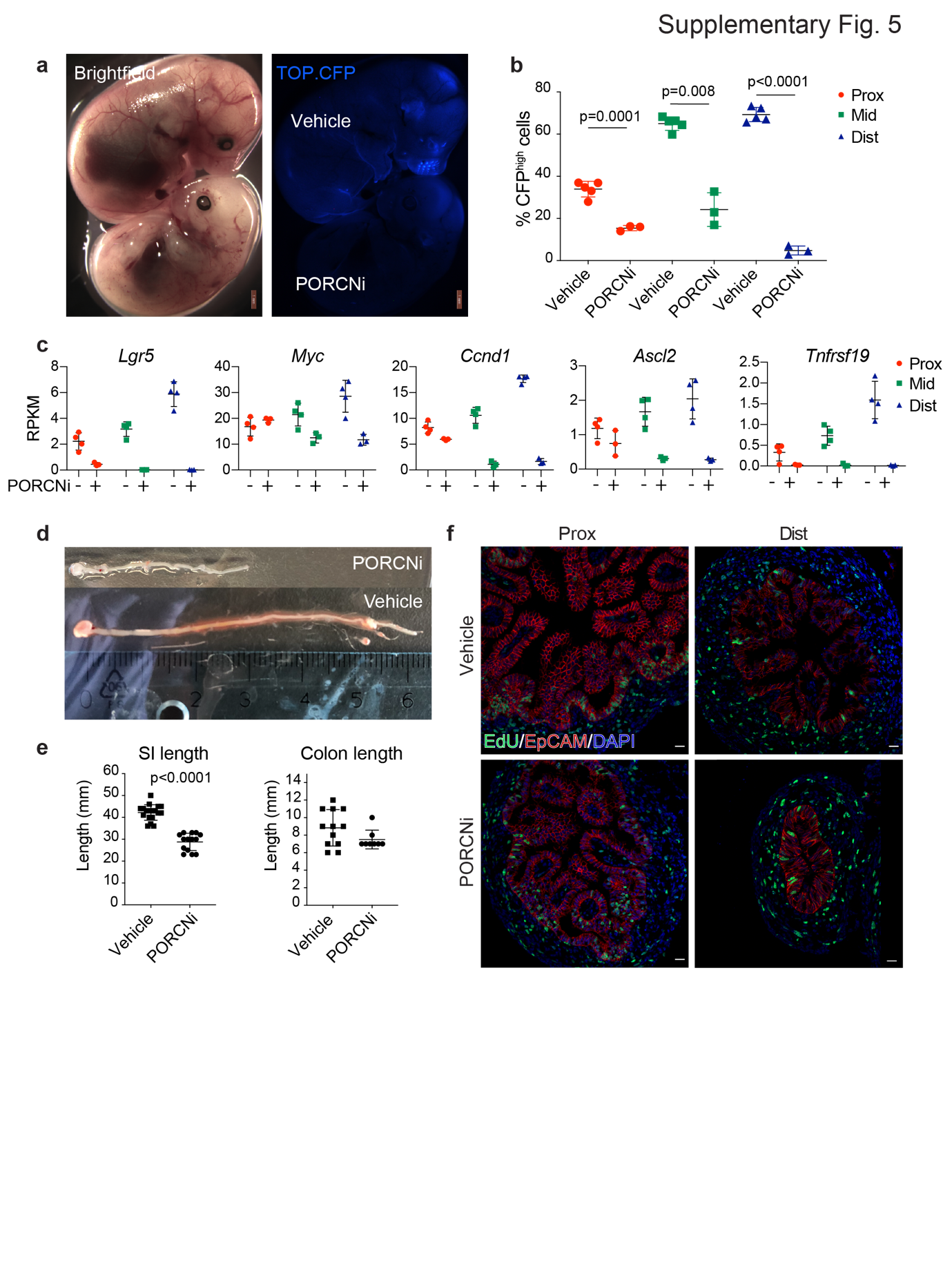


**Supplementary Fig. 5: Characterization of the effects of PORCN inhibitor during fetal development. a,** E16.5 Top.CFP embryos treated with vehicle (left) or PORCNi (right). Scale bar, 1 mm. **b,** Quantification of flow cytometry data depicting the reduction of CFP^+^ cells (DAPI^-^/CD31^-^/CD45^-^/EpCAM^+^) upon PORCN inhibition. **c,** RNA-seq data for the known Wnt target genes *Lgr5, Myc, Ccnd1, Ascl2, Tnfrsf19* with and without PORCN inhibition. **d,** Overview of E16.5 gut tube in vehicle and PORCN inhibitor treated animals. **e,** Quantification of SI and colon length in vehicle and PORCNi treated animals. **f,** Detection of EdU (green), EpCAM (red) and DAPI (blue) in proximal and distal regions in control and PORCNi samples. Scale bars, 20 μm. Results in b show mean ± s.d. from n = 5 (vehicle) and n = 3 (PORCN inhibition) biological replicates. In c data presented are means ± s.d. from n = 4 (control) and n = 3 (PORCN inhibition) biological replicates. Figure e presents mean ± s.d. of n = 18 (SI, vehicle), n = 14 (SI, PORCN inhibition), n = 12 (colon, vehicle) and n = 8 (colon, PORCN inhibition) biological replicates. In b and e statistical significance was assessed using two-tailed Welch’s t-test.

**
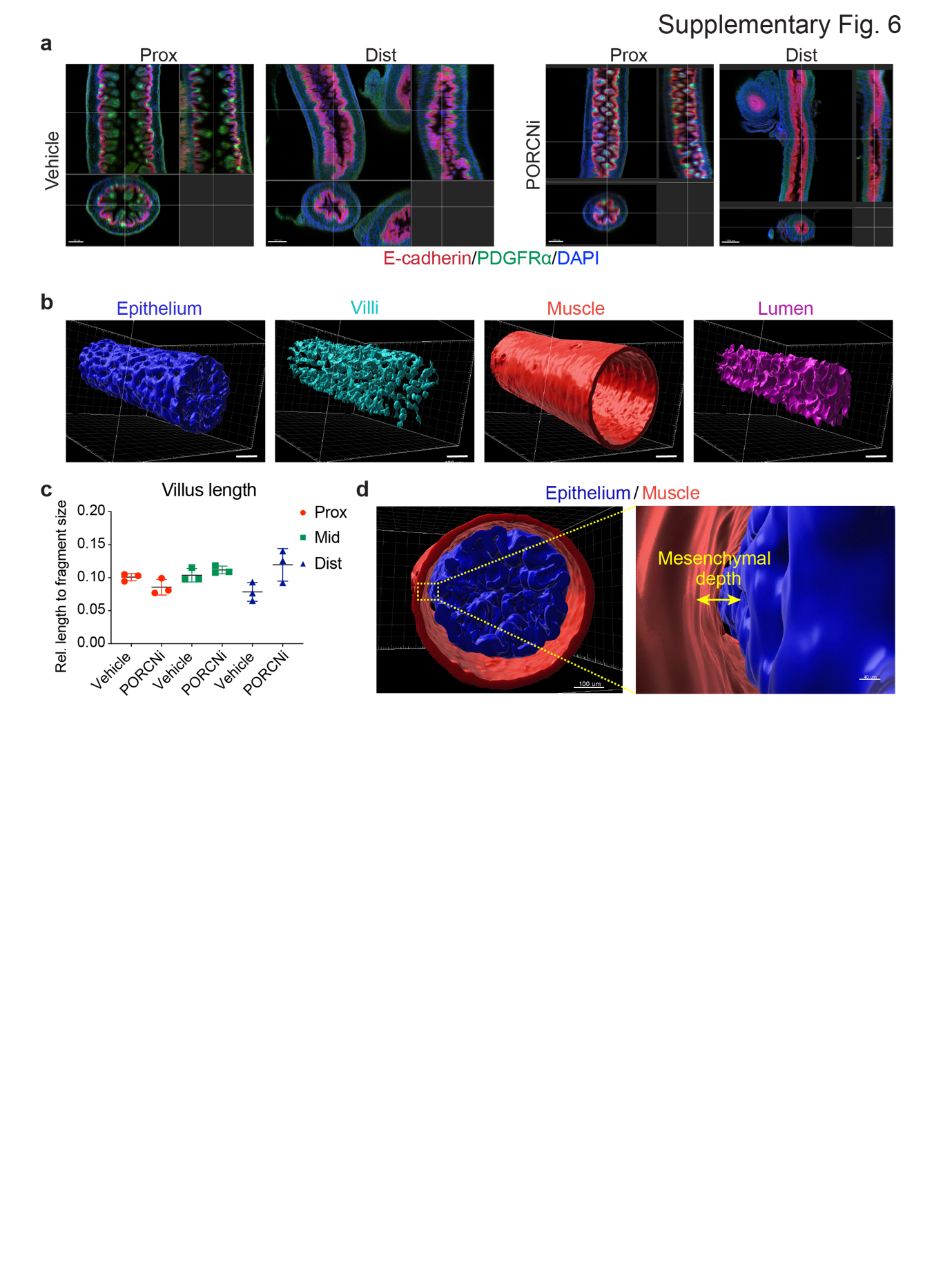
**

**Supplementary Fig. 6: Strategy for quantification of morphological features in the intestine. a,** 3D-whole mount immunostaining of E-cadherin (red), DAPI (blue) and PDGFRa (green) in vehicle and PORCNi treated animals. Displayed planes are xy, yz, and xz, respectively. Scale bars, 100 μm. **b,** Surface renderings of Ilastik-processed probability maps depicting epithelium (blue), villi (cyan), muscle (red) and lumen (purple). Scale bars, 100 μm. **c,** Quantification of villus length in vehicle and PORCNi treated animals. **d,** Strategy for measuring the depth of the mesenchyme. Epithelium: blue; muscle: red. Scale bars, 100 μm. Data in a and b are representative of n = 3 independent experiments. Figure c shows mean ± s.d. from n = 3 independent experiments.

**
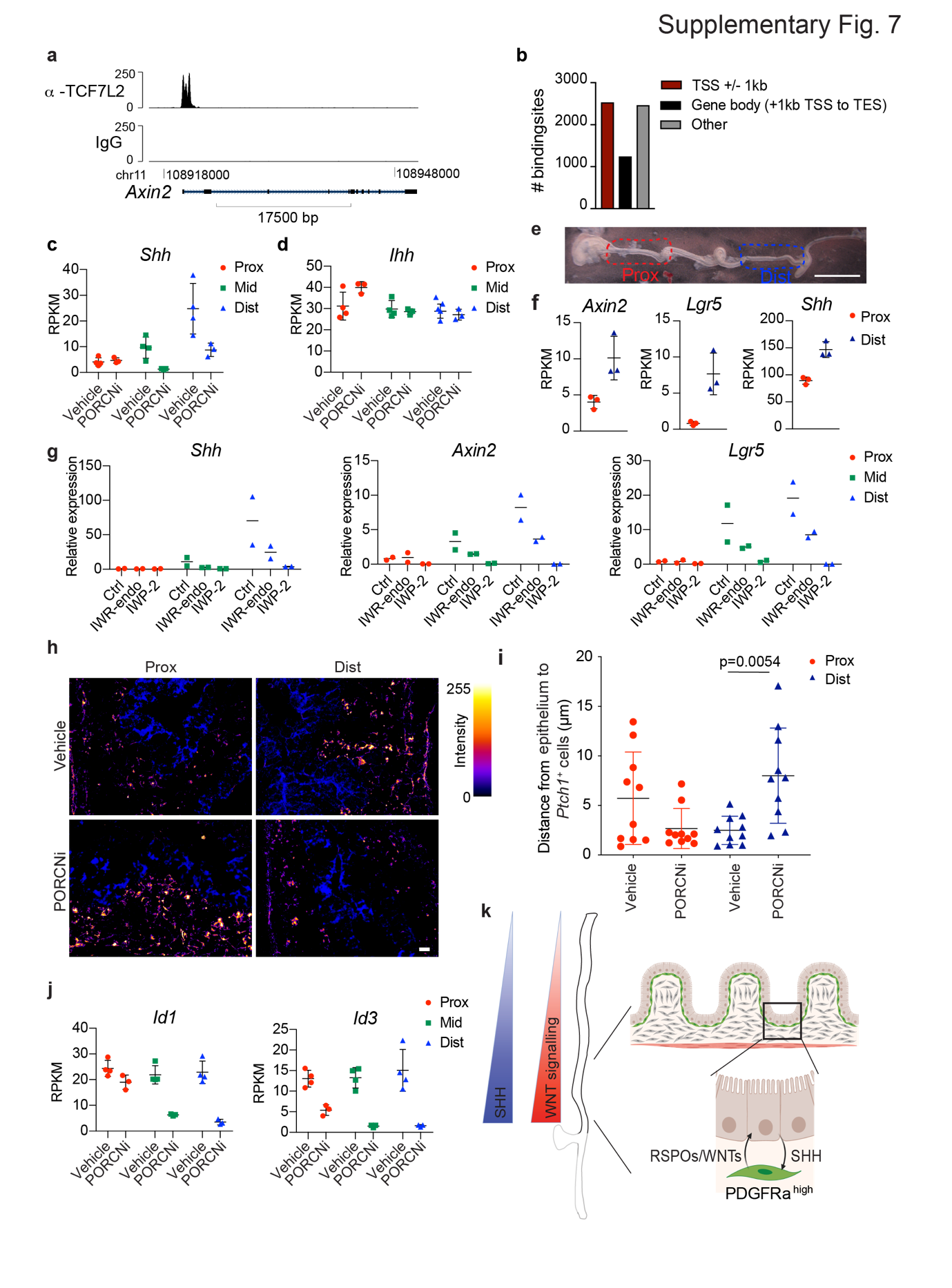
**

**Supplementary Fig. 7: Shh is a direct target for Wnt signalling. a,** ChIP-seq track depicting TCF7L2 coverage at the *Axin2* locus. **b,** Diagram illustrating the distribution of TCF7L2 peaks with regards to TSSs (transcription start site) and gene bodies. TES: transcription end site. **c-d,** *Shh* (c) and *Ihh* (d) expression levels in RNA-seq analyses of PORCNi and vehicle treated EpCAM^+^ cells. **e,** Overview of E13.5 SI and the division into proximal (prox) and distal (dist) regions. Scale bar, 200 μm**. f,** RNA-seq results for *Axin2*, *Lgr5* and *Shh,* from sorted EpCAM cells isolated from proximal and distal regions at E13.5. Note that only *Lgr5* is called as differentially expressed with the threshold of log2(fold change) > 0.5 and FDR < 0.05. **g,** qPCR analysis for *Shh, Axin2* and *Lgr5* from established organoid cultures generated from the indicated regions and cultured in ENR and treated with Vehicle, 10 μM IWR-1endo or 5 μM IWP-2 for 24h**. h,** In situ hybridisation for *Ptch1* (fire colour scheme) and *EpCAM* (blue) on proximal and distal regions in control and PORCNi samples. Images are deconvoluted from chromogenic results. Scale bar, 10 μm. **i,** Quantification of distance between *Ptch1*^+^ and *EpCAM*^+^ cluster. **j,** RNA-seq data for BMP target genes *Id1* and *Id3* with and without PORCN inhibition. Figure c, d and j show RNA-seq data from n = 4 (control) and n = 3 (PORCNi) and figure f for n = 3 biological replicates as mean ± s.d. Figure g shows mean for n = 2 independent experiments. Results in h are representative of n = 2 biological replicates, and results in i are a summary of images analysed from 2 independent experiments where 5 measurements were calculated per experiment. Significance was assessed using a two-tailed Welch’s t test. **k,** Schematic summary indicating the observed gradients of SHH and Wnt signalling with highest activity in the distal part of the small intestine. Here, RSPONDINs/WNTs secreted from the subepithelial PDGFRa^high^ (green) mesenchyme activate Wnt signalling in the epithelium leading to expression and secretion of SHH from the epithelium. This crosstalk promotes villus formation and shapes the developing small intestine. Created with [BioRender.com](http://BioRender.com).

|  | PROX | | | | | | MID | | | | | | DIST | | | | | |
| --- | --- | --- | --- | --- | --- | --- | --- | --- | --- | --- | --- | --- | --- | --- | --- | --- | --- | --- |
|  | CTRL | | | PORCNi | | | CTRL | | | PORCNi | | | CTRL | | | PORCNi | | |
|  | 1 | 2 | 3 | 1 | 4 | 3 | 1 | 2 | 3 | 2 | 3 | 4 | 1 | 2 | 3 | 1 | 4 | 3 |
| 1 | 54,29 | 52,05 | 52,50 | 55,59 | 18,15 | 52,87 | 54,81 | 27,65 | 27,88 | 52,32 | 57,34 | 38,62 | 60,07 | 80,12 | 65,13 | 31,58 | 37,40 | 31,16 |
| 2 | 53,24 | 31,68 | 24,15 | 41,77 | 50,62 | 51,90 | 34,44 | 49,57 | 40,36 | 31,89 | 33,42 | 46,51 | 66,38 | 70,93 | 83,84 | 28,71 | 29,56 | 38,84 |
| 3 | 57,49 | 52,39 | 37,08 | 51,36 | 41,19 | 44,47 | 54,81 | 28,41 | 42,95 | 53,76 | 37,01 | 49,52 | 86,30 | 63,36 | 77,38 | 30,14 | 44,25 | 33,68 |
| 4 | 57,49 | 46,78 | 28,88 | 46,53 | 57,76 | 52,20 | 38,83 | 37,34 | 34,00 | 33,32 | 47,91 | 30,57 | 66,90 | 64,89 | 56,47 | 29,73 | 40,72 | 39,81 |
| 5 | 71,05 | 62,98 | 49,30 | 38,91 | 31,45 | 54,60 | 42,86 | 25,63 | 30,06 | 54,76 | 38,34 | 31,70 | 76,61 | 56,91 | 59,19 | 32,52 | 46,15 | 34,90 |
| 6 | 50,10 | 37,17 | 29,97 | 46,36 | 61,98 | 46,44 | 39,28 | 47,73 | 44,59 | 42,28 | 28,97 | 24,25 | 83,84 | 56,91 | 70,21 | 32,16 | 41,26 | 46,56 |
| 7 | 71,38 | 67,75 | 46,89 | 43,57 | 19,20 | 26,72 | 78,85 | 35,87 | 27,58 | 46,43 | 24,17 | 50,34 | 70,09 | 52,85 | 72,48 | 42,17 | 40,28 | 40,14 |
| 8 | 59,68 | 46,25 | 30,58 | 40,05 | 53,62 | 58,45 | 23,33 | 41,99 | 68,48 | 26,89 | 34,94 | 38,25 | 85,47 | 62,18 | 70,39 | 39,22 | 41,57 | 59,09 |
| 9 | 60,16 | 49,06 | 28,82 | 46,33 | 16,01 | 44,68 | 47,90 | 27,88 | 27,80 | 49,44 | 35,35 | 37,81 | 59,70 | 39,75 | 61,97 | 50,42 | 41,46 | 43,50 |
| 10 | 55,15 | 46,68 | 35,22 | 43,44 | 50,93 | 48,22 | 34,84 | 48,07 | 51,88 | 32,43 | 29,05 | 51,42 | 82,59 | 49,62 | 81,91 | 43,80 | 38,94 | 52,68 |
| 11 | 64,31 | 22,63 | 39,76 | 51,75 | 49,67 | 39,27 | 35,42 | 29,73 | 33,35 | 35,75 | 27,98 | 32,00 | 61,06 | 66,56 | 67,74 | 45,82 | 42,60 | 35,45 |
| 12 | 57,76 | 40,85 | 24,66 | 50,99 | 39,06 | 45,30 | 23,38 | 50,10 | 60,58 | 36,52 | 36,36 | 35,90 | 81,63 | 65,77 | 69,12 | 40,13 | 43,54 | 56,16 |
| 13 | 68,39 | 44,21 | 60,46 | 35,49 | 28,98 | 50,10 | 43,04 | 36,46 | 24,57 | 34,33 | 34,59 | 41,16 | 66,85 | 76,21 | 69,74 | 39,71 | 43,27 | 37,98 |
| 14 | 55,41 | 53,63 | 33,57 | 49,83 | 44,33 | 50,08 | 48,01 | 56,02 | 48,27 | 32,82 | 49,10 | 28,06 | 91,08 | 71,83 | 62,03 | 36,47 | 47,03 | 46,55 |
| 15 | 77,68 | 41,93 | 31,68 | 62,49 | 24,93 | 55,39 | 56,91 | 34,39 | 28,71 | 42,03 | 23,09 | 28,99 | 68,48 | 95,36 | 101,70 | 46,28 | 44,91 | 41,97 |
| 16 | 51,00 | 47,62 | 50,07 | 38,59 | 56,36 | 63,76 | 25,25 | 49,66 | 52,50 | 22,31 | 34,23 | 53,17 | 82,69 | 64,85 | 55,01 | 42,11 | 39,76 | 45,56 |
| 17 | 68,03 | 30,55 | 48,01 | 65,51 | 28,88 | 37,88 | 56,76 | 42,06 | 27,45 | 49,08 | 20,45 | 31,47 | 61,49 | 63,97 | 56,32 | 47,14 | 50,51 | 41,63 |
| 18 | 63,23 | 28,75 | 40,04 | 41,13 | 38,21 | 55,29 | 31,71 | 45,54 | 54,88 | 18,63 | 33,69 | 36,56 | 80,72 | 65,53 | 64,36 | 46,23 | 30,58 | 39,32 |
| 19 | 55,64 | 29,10 | 55,23 | 42,89 | 29,90 | 35,03 | 47,47 | 44,13 | 24,08 | 51,11 | 31,52 | 36,09 | 75,15 | 65,38 | 78,24 | 56,92 | 43,91 | 56,96 |
| 20 | 67,20 | 43,49 | 32,89 | 59,73 | 50,46 | 65,37 | 29,06 | 49,53 | 53,52 | 25,61 | 34,96 | 28,57 | 54,12 | 63,88 | 73,97 | 56,56 | 26,64 | 37,85 |
|  | 60,93 | 43,78 | 38,99 | 47,62 | 39,58 | 48,90 | 42,35 | 40,39 | 40,17 | 38,59 | 34,62 | 37,55 | 73,06 | 64,84 | 69,86 | 40,89 | 40,72 | 42,99 |

**Supplementary table 1: Distances measured between epithelium and muscle.**

| GeneID | Symbol | GeneID | Symbol | GeneID | Symbol |
| --- | --- | --- | --- | --- | --- |
| 497097 | Xkr4 | 20609 | Sstr5 | 74318 | Hopx |
| 404634 | H2afy2 | 66443 | Tnfaip8l1 | 319616 | 5930412G12Rik |
| 67405 | Nts | 381122 | Capn13 | 93897 | Fzd10 |
| 108030 | Lin7a | 381157 | Greb1l | 243277 | Adgrd1 |
| 14160 | Lgr5 | 11829 | Aqp4 | 68895 | Rasl11a |
| 14401 | Gabrb2 | 71373 | Prr16 | 13426 | Dync1i1 |
| 319767 | Atp10b | 107221 | Ffar4 | 330267 | Thsd7a |
| 15416 | Hoxb8 | 22352 | Vim | 213391 | Rassf4 |
| 19049 | Ppp1r1b | 227632 | Kcnt1 | 26365 | Ceacam1 |
| 66599 | Rdm1 | 74016 | Phf19 | 26367 | Ceacam2 |
| 217212 | Pyy | 14526 | Gcg | 170711 | Otud7a |
| 12006 | Axin2 | 13661 | Ehf | 100038419 | E330027M22Rik |
| 12810 | Coch | 20192 | Ryr3 | 634825 | Gm14851 |
| 20963 | Syk | 19261 | Sirpa | 13218 | Defa29 |
| 218763 | Lrrc3b | 109222 | Rarres1 | 100505096 | Defa33 |
| 70561 | Txndc16 | 57257 | Vav3 | 665927 | Gm7849 |
| 23888 | Gpc6 | 76131 | Depdc1a | 68009 | Defa20 |
| 223433 | Otulinl | 252838 | Tox | 100041890 | Defa32 |
| 18383 | Tnfrsf11b | 384009 | Glipr2 | 665956 | Gm7861 |
| 100504191 | Gm16576 | 23919 | Insl5 | 235106 | Ntm |
| 56233 | Hdac7 | 66805 | Tspan1 | 24046 | Scn11a |
| 117606 | Boc | 16765 | Stmn1 | 236781 | Gpr119 |
| 12837 | Col8a1 | 20423 | Shh | 57385 | P2ry4 |
| 268902 | Robo2 | 81840 | Sorcs2 |  |  |
| 26559 | Hunk | 14181 | Fgfbp1 |  |  |

**Supplementary table 2: Overlap of Wnt-dependent distal genes.**

|  |  |  |  |  |
| --- | --- | --- | --- | --- |
| **** |  |  |  |  |
| **Supplementary table 3: List of antibodies used.** |  |  |  |  |
