# Supplementary material for: Mesenchymal-epithelial crosstalk shapes intestinal regionalisation via Wnt and Shh signalling
Source: Nat Commun. 2022 Feb 7;13:715. doi: 10.1038/s41467-022-28369-7 (PMC8821716; doi:10.1038/s41467-022-28369-7)
Supplement: Supplementary file 3 — Description of Additional Supplementary Files [file 41467_2022_28369_MOESM3_ESM.pdf]

Description of Additional Supplementary Files

File name: Supplementary Data 1

Description: DE genes in CD29_high versus PDGFRa_med comparisons

File name: Supplementary Data 2

Description: GO analyses of the CD29_high signature

File name: Supplementary Data 3

Description: DE genes for PDGFRa_high, PDGFRa_med and $EpCAM^{+}$ populations

File name: Supplementary Data 4

Description: GO analyses of PDGFRa_high and PDGFRa_med signatures

File name: Supplementary Data 5

Description: DE genes for control versus PORCNi treated $EpCAM^{+}$ cells

File name: Supplementary Data 6

Description: Annotated TCF7L2 ChIP-seq peaks

File name: Supplementary Data 7

Description: DE genes for E13.5 $EpCAM^{+}$ populations

File name: Supplementary Movie 1

Description: Visualisation of morphological features in the fetal intestine. Segmentation of tissue into lumen (magenta), epithelium (blue), villi (cyan), and muscle (red) subsequent to 3D imaging, probability map building and surface rendering (see Methods). Scale bar, 50 µm.

File name: Supplementary Movie 2

Description: Ex vivo cultured intestine undergoes villus formation. E13.5 fetal whole intestine isolated from PDGFRaCreERT2;mTmG animal and cultured for 48h in the presence of DMSO depicting the formation of PDGFRa+ clusters and peristaltic movements.
