# Supplementary material for: Mesenchymal-epithelial crosstalk shapes intestinal regionalisation via Wnt and Shh signalling
Source: Nat Commun. 2022 Feb 7;13:715. doi: 10.1038/s41467-022-28369-7 (PMC8821716; doi:10.1038/s41467-022-28369-7)
Supplement: Supplementary file 13 — Reporting Summary [file 41467_2022_28369_MOESM13_ESM.pdf]

nature research

Corresponding author(s): Kim B. Jensen

Last updated by author(s): Dec 13, 2021

Reporting Summary

Nature Research wishes to improve the reproducibility of the work that we publish. This form provides structure for consistency and transparency in reporting. For further information on Nature Research policies, see our Editorial Policies and the Editorial Policy Checklist.

Statistics

For all statistical analyses, confirm that the following items are present in the figure legend, table legend, main text, or Methods section.

n/a	Confirmed	
☐	☒	The exact sample size (*n*) for each experimental group/condition, given as a discrete number and unit of measurement
☐	☒	A statement on whether measurements were taken from distinct samples or whether the same sample was measured repeatedly
☐	☒	The statistical test(s) used AND whether they are one- or two-sided<br>*Only common tests should be described solely by name; describe more complex techniques in the Methods section.*
☒	☐	A description of all covariates tested
☒	☐	A description of any assumptions or corrections, such as tests of normality and adjustment for multiple comparisons
☐	☒	A full description of the statistical parameters including central tendency (e.g. means) or other basic estimates (e.g. regression coefficient) AND variation (e.g. standard deviation) or associated estimates of uncertainty (e.g. confidence intervals)
☐	☒	For null hypothesis testing, the test statistic (e.g. *F*, *t*, *r*) with confidence intervals, effect sizes, degrees of freedom and *P* value noted<br>*Give P values as exact values whenever suitable.*
☒	☐	For Bayesian analysis, information on the choice of priors and Markov chain Monte Carlo settings
☒	☐	For hierarchical and complex designs, identification of the appropriate level for tests and full reporting of outcomes
☒	☐	Estimates of effect sizes (e.g. Cohen's *d*, Pearson's *r*), indicating how they were calculated

Our web collection on statistics for biologists contains articles on many of the points above.

Software and code

Policy information about availability of computer code

Data collection	Leica LAS X , BD FACSDiva v9.0, bcl2fastq conversion software (Illumina)
Data analysis	Ilastik version 1.3.3post3, Imaris 9.6, ImarisViewer 9.5.1, Kaluza 2.1.2, ImageJ 2.0.0-rc-59/1.51n, NDP.view2 U12388-01, FastQ Screen (Galaxy Version 0.1.3), FastQC (Galaxy Version 0.72), Trim Galore! (Galaxy version 0.4.2), Bowtie for Illumina (Galaxy version 1.2.0), Rmdup function (Galaxy Version 2.0.1), MACS2 callpeak (Galaxy Version 2.1.1.20160309.6), Easeq (http:easeq.net, version 1.111) as well as the following packages in R: Rsubread (2.0), featureCounts (1.6.3), edgeR (3.28), limma (3.42), pheatmap (version 1.0.10), and eulerr (version 6.0.0).

For manuscripts utilizing custom algorithms or software that are central to the research but not yet described in published literature, software must be made available to editors and reviewers. We strongly encourage code deposition in a community repository (e.g. GitHub). See the Nature Research guidelines for submitting code & software for further information.

Data

Policy information about availability of data

All manuscripts must include a data availability statement. This statement should provide the following information, where applicable:

- Accession codes, unique identifiers, or web links for publicly available datasets
- A list of figures that have associated raw data
- A description of any restrictions on data availability

The RNA-seq and ChIP-seq data generated in this study have been deposited in the NCBI's Gene Expression Omnibus (GEO) database.

Field-specific reporting

Please select the one below that is the best fit for your research. If you are not sure, read the appropriate sections before making your selection.

☒ Life sciences ☐ Behavioural & social sciences ☐ Ecological, evolutionary & environmental sciences

For a reference copy of the document with all sections, see nature.com/documents/nr-reporting-summary-flat.pdf

Life sciences study design

All studies must disclose on these points even when the disclosure is negative.

Sample size	Sample sizes were estimated on the basis of previous studies using similar methods and analyses that are widely published (e.g. Yui et al., 2018, Cell Stem Cell; Huels et al., 2018, Nature Communication).
Data exclusions	No data were excluded
Replication	Experimental data was replicated using at least 3 independent biological samples (stated in the figure legends), except for the in situ hybridisation, where 2 biological replicates were assessed
Randomization	For mouse studies randomized cohorts including both male and female animals were distributed in an unblinded manner into the experimental time points for analysis.
Blinding	No blinding was used in these experiments because the same investigator designed and conducted the experiments including the appropriate controls.

Reporting for specific materials, systems and methods

We require information from authors about some types of materials, experimental systems and methods used in many studies. Here, indicate whether each material, system or method listed is relevant to your study. If you are not sure if a list item applies to your research, read the appropriate section before selecting a response.

Materials & experimental systems

n/a	Involved in the study	
☐	☒	Antibodies
☒	☐	Eukaryotic cell lines
☒	☐	Palaeontology and archaeology
☐	☒	Animals and other organisms
☒	☐	Human research participants
☒	☐	Clinical data
☒	☐	Dual use research of concern

Methods

n/a	Involved in the study	
☐	☒	ChIP-seq
☐	☒	Flow cytometry
☒	☐	MRI-based neuroimaging

Antibodies

Antibodies used

E-Cadherin (BD Bioscience, 610181, Clone 36)
TCF7L2 (clone C48H11, #2569, Cell Signaling)
Lysozyme 1 (DAKO, A0099, EC.3.2.1.17)
CD140a, PDGF Receptor a (ThermoFisher, #25-1401-82, Clone APA5)
EpCAM (ThermoFisher, #17-5791-82, Clone G8.8)
CD29 (ThermoFisher, #46-0291-82, Clone HMb1-1)
CD45 (BD Bioscience, 550994, Clone 30-F11)
CD31 (BD Bioscience, 562861, Clone MEC 13.3)
LRIG1 PE-conjugated Antibody (R&D Systems, FAB3688P)
Nephronectin (Gift from Hironobu Fujiwara, Clone CUK1192)
Laminin a5 (Gift from Lydia Sorokin, Clone 504)
Laminin b1 (Gift from Jeff Miner, Clone 1065)
Shh (RM0128-4A37, ab86462, Abcam)
Alexa Flour 488 Donkey anti Rabbit IgG (H+L) ThermoFisher A-21206
Alexa Fluor 488 Donkey anti Rat IgG (H+L) ThermoFisher A-21208
Alexa Fluor 555 Goat anti mouse IgG2a ThermoFisher A-21137

Validation

E-Cadherin, Jaksits et al., 1999, CD34+ Cell-Derived CD14+ Precursor Cells Develop into Langerhans Cells in a TGF-β1-Dependent Manner, J Immunol
TCF7L2, ChIP data and 13 references for ChIP on company website: https://www.cellsignal.com/products/primary-antibodies/tcf4-tcf7l2-c48h11-rabbit-mab/2569

Lysozyme 1, Meister et al., 1980, Malignant histiocytosis. Immunohistochemical characterization on paraffin-embedded tissue., Virchows Arch A Path Anat Histol
CD140a (PDGFRa), 25 references on company website: https://www.thermofisher.com/antibody/product/CD140a-PDGFRA-Antibody-clone-APA5-Monoclonal/25-1401-82
EpCAM, 56 references on company website: https://www.thermofisher.com/antibody/product/CD326-EpCAM-Antibody-clone-G8-8-Monoclonal/17-5791-82
CD29, 24 references on company website: https://www.thermofisher.com/antibody/product/CD29-Integrin-beta-1-Antibody-clone-eBioHMb1-1-HMb1-1-Monoclonal/46-0291-82
CD45, Shapiro HM. Practical Flow Cytometry, 3rd Edition. New York: Wiley-Liss, Inc; 1995; :280-281.
LRIG1 , doi: 10.1038/ncb2464.
Nephronectin, DOI: 10.7554/eLife.38883
CD31, Vanzulli S, Gazzaniga S, Braidot MF, et al. Detection of endothelial cells by MEC 13.3 monoclonal antibody in mice mammary tumors. Biocell. 1997; 21(1):39-46.
Laminin a5, Simo et al, 1991, Changes in the expression of laminin during intestinal development, Development
Laminin b1, doi: 10.1046/j.0014-2956.2001.02663.x.
Shh, Williamson et al., 2019, doi: 10.1242/dev.179523

Animals and other organisms

Policy information about studies involving animals; ARRIVE guidelines recommended for reporting animal research

Laboratory animals

Mus musculus aged between 8 and 16 weeks were used as animal model. Fetal tissues was isolated from at E13.5 or E16.5.
The strains indicated below were used:
-C57BL/6J mice purchased from Taconic
-TOP.CFP
-PDGFRaCreERT2
-mTmG

Males and females were used for the study. All animals were housed in SPF (specific pathogen free) animal facilities, in either open or individually ventilated cages always with companion mice, and cages were placed under a 12hr light-dark cycle. Food and water were provided ad libitum. The room temperature for mice was 22°C and the relative humidity was kept between 45% and 65%.

Wild animals

No wild animals were used

Field-collected samples

No field-collected samples were used

Ethics oversight

The National Animal Inspectorate in Denmark reviewed and approved all animal procedures (Permit numbers 2017-15-0201-01381 and 2018-15-0201-01569)

Note that full information on the approval of the study protocol must also be provided in the manuscript.

ChIP-seq

Data deposition

☒ Confirm that both raw and final processed data have been deposited in a public database such as GEO.

☒ Confirm that you have deposited or provided access to graph files (e.g. BED files) for the called peaks.

Data access links
May remain private before publication.

The RNA-seq and ChIP-seq data generated in this study have been deposited in the NCBI's Gene Expression Omnibus (GEO) database under accession code GSE183671, [https://www.ncbi.nlm.nih.gov/geo/query/acc.cgi?acc=GSE183671].

Files in database submission

ChIP-seq: TCF7L2.fastqsanger, IgG.fastqsanger, TCF7L2.wig, IgG.wig, TCF7L2_vs_IgG_peaks.tabular.
RNA-seq: 54 fastq-files and GSE183532_RPKMs.xlsx

Genome browser session
(e.g. UCSC)

https://genome-euro.ucsc.edu/s/leonorrib/mm10_chipseq_peaks

Methodology

Replicates

One ChIP-seq replicate (TCF7L2-antibody and IgG control) is submitted.

Sequencing depth

Total number of reads: anti-TCF7L2 (20871783), anti-IgG (39469040). Uniquely mapped reads: anti-TCF7L2 (4343120), IgG (5797120). Length of reads: 76 bp. Single-end.

Antibodies

For ChIP-seq, we used a rabbit monoclonal antibody recognizing TCF7L2 (clone C48H11, #2569, Cell Signaling).

Peak calling parameters

Concatenated reads were trimmed using Trim Galore! (Galaxy version 0.4.2) with trimming of "Illumina Universal" adaptor sequences and standard settings except N=2 (remove 2 bp from the 3' end), and N=25 (Discard reads that became shorter than length 25). Trimmed reads were mapped to mm10 canonical (April 2020) using Map with Bowtie for Illumina (Galaxy version 1.2.0) with standard settings except m=1 (supress all alignments if >1 exist). After SAM-to-BAM conversion, duplicate reads were removed using the RmDup function (Galaxy Version 2.0.1). Peak calling was performed using MACS2 callpeak (Galaxy Version 2.1.1.20160309.6) using the IgG ChIP sample as negative control and standard settings except effective genome size=M. musculus (1.87e9). BAM files and MACS2 output files were imported into Easeq (http://easeq.net, (Lerdrup, M. et al. Nat Struct Mol Biol 23, 349-357, doi:10.1038/

	nsmb.3180 2016)) which was used for the generation of plots and peak-annotation to mm10 Refseq genes (downloaded from UCSC, May 2020).
Data quality	Initial quality of fastq files were assessed using FastQ Screen (Galaxy Version 0.1.3) and FastQC (Galaxy Version 0.72). The quality of peak-finding was confirmed by data visualization (using heatmap and ChIP-seq tracks) which showed specific enrichment at expected sites comparing the anti-TCF7L2 and IgG (negative control) datasets. 6238 peaks were identified using MACS2 callpeak (Galaxy Version 2.1.1.20160309.6) with standard parameters.
Software	Data was collected using bcl2fastq conversion software (Illumina). Fastq files were analysed using Galaxy (https://galaxyproject.org) and Easeq (http://easeq.net) (Lerdrup, M. et al. Nat Struct Mol Biol 23, 349-357, doi:10.1038/nsmb.3180 2016).

Flow Cytometry

Plots

Confirm that:

☒ The axis labels state the marker and fluorochrome used (e.g. CD4-FITC).

☒ The axis scales are clearly visible. Include numbers along axes only for bottom left plot of group (a 'group' is an analysis of identical markers).

☒ All plots are contour plots with outliers or pseudocolor plots.

☒ A numerical value for number of cells or percentage (with statistics) is provided.

Methodology

Sample preparation	Fetal small intestine from E16.5 fetuses was dissected and incubated with collagenase (Sigma) (125 µg/ml) in 0.1% BSA in PBS for 45 min at 37 °C and subjected to vigorous pipetting every 15 min using a P1000 pipette. Released cells were pelleted and resuspended in PBS supplemented with 1% BSA and incubated with fluorescent-conjugated primary antibodies for 15 min at room temperature. After washing, 1 µM DAPI was added to the cell suspension to facilitate exclusion of dead cells by.
Instrument	FACSAria III (BD Bioscience)
Software	BD FACSDiva 9,0<br>Kaluza 2.1.2
Cell population abundance	Purity of the sorted populations were determined by re-sorting experiments.
Gating strategy	After removing cell clumps, debris and dead cells relevant cell populations were gated based on single stained controls or fluorochrome-minus-one controls in case fluorochrome combinations required significant compensation.

☒ Tick this box to confirm that a figure exemplifying the gating strategy is provided in the Supplementary Information.
